# Brain Oscillation Entrainment by Perceptible and Non-perceptible Rhythmic Light Stimulation

**DOI:** 10.3389/fnrgo.2021.646225

**Published:** 2021-04-14

**Authors:** Katharina Lingelbach, Alexander M. Dreyer, Isabel Schöllhorn, Michael Bui, Michael Weng, Frederik Diederichs, Jochem W. Rieger, Ina Petermann-Stock, Mathias Vukelić

**Affiliations:** ^1^Fraunhofer Institute for Industrial Engineering, Human-Technology Interaction, Stuttgart, Germany; ^2^Department of Psychology, European Medical School, University of Oldenburg, Oldenburg, Germany; ^3^Centre for Chronobiology, Psychiatric Hospital of the University of Basel, Basel, Switzerland; ^4^Transfaculty Research Platform Molecular and Cognitive Neurosciences, University of Basel, Basel, Switzerland; ^5^Volkswagen AG, Group Innovation, Wolfsburg, Germany

**Keywords:** neuroergonomics, steady-state visual evoked potentials, non-perceptible stimulation, attention, entrainment of oscillations, functional connectivity, vehicle interior

## Abstract

**Objective and Background:** Decades of research in the field of steady-state visual evoked potentials (SSVEPs) have revealed great potential of rhythmic light stimulation for brain–computer interfaces. Additionally, rhythmic light stimulation provides a non-invasive method for entrainment of oscillatory activity in the brain. Especially effective protocols enabling non-perceptible rhythmic stimulation and, thereby, reducing eye fatigue and user discomfort are favorable. Here, we investigate effects of (1) perceptible and (2) non-perceptible rhythmic light stimulation as well as attention-based effects of the stimulation by asking participants to focus (a) on the stimulation source directly in an overt attention condition or (b) on a cross-hair below the stimulation source in a covert attention condition.

**Method:** SSVEPs at 10 Hz were evoked with a light-emitting diode (LED) driven by frequency-modulated signals and amplitudes of the current intensity either below or above a previously estimated individual threshold. Furthermore, we explored the effect of attention by asking participants to fixate on the LED directly in the overt attention condition and indirectly attend it in the covert attention condition. By measuring electroencephalography, we analyzed differences between conditions regarding the detection of reliable SSVEPs via the signal-to-noise ratio (SNR) and functional connectivity in occipito-frontal(-central) regions.

**Results:** We could observe SSVEPs at 10 Hz for the perceptible and non-perceptible rhythmic light stimulation not only in the overt but also in the covert attention condition. The SNR and SSVEP amplitudes did not differ between the conditions and SNR values were in all except one participant above significance thresholds suggested by previous literature indicating reliable SSVEP responses. No difference between the conditions could be observed in the functional connectivity in occipito-frontal(-central) regions.

**Conclusion:** The finding of robust SSVEPs even for non-intrusive rhythmic stimulation protocols below an individual perceptibility threshold and without direct fixation on the stimulation source reveals strong potential as a safe stimulation method for oscillatory entrainment in naturalistic applications.

## 1. Introduction

In the recent years, a rapidly growing number of studies investigated rhythmic light stimulation in order to evoke synchronized neuronal firing and, thus, entrain brain activity. Neurophysiological effects of such non-invasive brain stimulation can be investigated via the electroencephalography (EEG) and revealed great potential for brain–computer interface (BCI) applications due to their high signal-to-noise ratio (SNR; see Vialatte et al., 2010; Norcia et al., 2015 for reviews). Moreover, these stimulations are suggested to be a safe method to modulate brain activity with comparable effects as other non-invasive electric current stimulation methods (Zaehle et al., 2010; Notbohm and Herrmann, 2016; Notbohm et al., 2016). Several studies already reported psychophysiological effects of naturalistic light stimulations on subjective well-being, cognitive performance, and physical well-being, such as sleep quality (Terman and Terman, 2006; Gabel et al., 2015; Viola et al., 2015). Especially for safety-critical situations, possibilities to enhance alertness, concentration, performance, or a combination of them by modulating brain activity and neuronal functioning are appealing.

When stimulating the brain via a flickering light source, so-called steady-state visual evoked potentials (SSVEPs) are elicited in response to the train of rhythmic stimuli (Regan, 1966; see Norcia et al., 2015 for a review). These oscillatory brain responses lead to an increase in the amplitude at the same frequency as the stimulation. SSVEPs can be detected by comparing the power in the frequency stimulated at to the power in neighboring frequencies (Meigen and Bach, 1999; Norcia et al., 2015). The SSVEPs are most prominent in the visual cortex with rather narrow regional power modulation (Vialatte et al., 2010). Apart from regional modulations, Lithari et al. (2016) observed distributed large-scale entrainment via graph theoretical measures in functional cortical networks. They reported a frequency-unspecific reduction of density in the alpha band reflecting a disconnection of the visual cortex from the rest of the network. In addition, increased connectivity between the occipital cortex and precuneus was found at a high stimulation frequency with an exhibited resonance at 30 Hz. There are several theories concerning whether the SSVEP is strictly a local phenomenon or how it might propagate to more broadly distributed non-visual regions (Srinivasan et al., 2007; Vialatte et al., 2010; Lithari et al., 2016). Some research suggests that the SSVEP response involves multiple distributed cortical sources, including the parietal, temporal, frontal, and prefrontal cortices (Srinivasan et al., 2007; Yan and Gao, 2011; Lithari et al., 2016). Thereby, the stimulation frequency and physical properties of the stimulation seem to play a significant role in shaping the extent of the functional connectivity profiles measured in response to the rhythmic stimulation (Srinivasan et al., 2007; Lithari et al., 2016).

Regarding real-world application, Fan et al. (2014) introduced a promising approach to use SSVEPs in a hybrid BCI-based driver–vehicle interface for disabled individuals. Their destination selection system allows to select the destination of a ride in a laboratory and real driving environment via the combination of a P300-based selection component and SSVEP-based confirmation component in BCIs. The inclusion of the SSVEP-based confirmation component improved accuracy of the destination selection system. From a neuroergonomic and user experience perspective, however, two aspects of rhythmic light stimulations need to be considered: (1) The continuous exposure to the flickering light sources strains the sensory system and results in user discomfort (Ortner et al., 2011) as well as fatigue on a subjectively perceived and physical level (Cao et al., 2013). (2) Several studies suggest that strongest power modulations are evoked when the flickering light source is positioned in the center of the visual field and directly fixated (i.e., with focused attention; Herrmann, 2001; Ng et al., 2012; Ordikhani-Seyedlar et al., 2014), but this configuration is rather incompatible with the implementation in most everyday life environments and tasks due to its disturbing nature. Therefore, it is essential to explore suitable protocols characterized by (1) non-intrusiveness and increased user comfort (e.g., by using less perceptible flickering stimulations), and (2) feasibility regarding their integration (e.g., by exploring entrainment effects during a stimulation that does not require the direct fixation on the light source).

Previous literature suggests protocols with high frequent flickering (above 30 Hz) to be less perceptible and irritating than those with low frequent flickering (below 30 Hz; Lin et al., 2012; Sakurada et al., 2015 for 41, 43, and 45 vs. 61, 63, and 65 Hz). However, this advantage comes at the cost of decreased SSVEP power (Won et al., 2015). Further studies investigated frequency-modulated (FM) stimulations, since they allow for a less perceptible stimulation and, thus, decrease of potential eye fatigue and user discomfort (Dreyer and Herrmann, 2015; Dreyer et al., 2017). In FM stimulation protocols, a carrier frequency (typically a high frequency) is modulated by a second frequency, so-called modulation frequency. Thereby, SSVEPs are evoked at the difference of the chosen carrier and modulation frequency. Dreyer and Herrmann (2015) explored the subjective perceptibility of various carrier (20–100 Hz) and modulation (10–90 Hz) frequency combinations (20–10, 30–20,., 100–90 Hz) and reported reliable FM SSVEPs at 10 Hz. Especially, FM stimulations were subjectively rated as less perceptible in comparison to other stimulation protocols (Dreyer and Herrmann, 2015; Dreyer et al., 2017).

Rather sparse literature can be found on research investigating effects of covertly attended rhythmic light stimulation. This might be explained by reduced effects of the stimulation when participants indirectly fixate on it. Müller et al. (1998) examined the effect of spatial selective attention by presenting flickering LED stimuli in one visual field with similar stimuli in the opposite unattended visual field. They observed decreased SSVEP amplitudes for the spatially unattended stimuli (see also Hillyard et al., 1998; Müller and Hillyard, 2000; Müller et al., 2003). Ordikhani-Seyedlar et al. (2014) presented stimuli that were directly attended (overt) or indirectly attended (covert) either in the left or right visual field. In line with previous findings, the authors observed reduced SSVEP amplitudes in the covertly attended compared to the overtly attended stimulation condition. Interestingly, the power in the second harmonic response increased for the covertly attended stimulation. Walter et al. (2012) observed a significant decrease in SSVEP amplitudes for covertly attended stimuli along with a difference in the topographical distribution: For overtly attended stimuli, largest amplitudes were at electrodes over centro-occipital regions. While covertly attended stimuli were associated with largest amplitudes at electrodes over contralateral parieto-occipital regions. Dreyer et al. (2017) aimed at comparing SSVEPs for fixated and non-fixated flickering stimuli presented in a circle either above, below, left, or right of a central fixation point. The authors reported that no reliable SSVEPs could be evoked in the covert attention condition. Another study used two series of random disk search arrays as stimuli, each superimposed on two concentric color-coded circles (Ding et al., 2006). They updated the two series of arrays independently; one at a fixed frequency and the other randomly according to a white noise distribution. Participants were instructed to observe a circle and detect a target that occasionally appeared in a random disk array in the observed circle. Inconsistent with previous described studies, Ding et al. (2006) observed a reversed effect of spatial attention with higher SSVEP amplitudes in the unattended conditions at flicker frequencies in the lower alpha band (8–10 Hz). However, the reversed effect was not observable in other conditions (see discussion in Toffanin et al., 2009).

This previous research leaves two questions important for potential practical applications open: namely, the effects of attention and perceptibility and their interaction during rhythmic light stimulation. Therefore, we were interested whether (1) participants had to fixate on the light source (with overt attention) compared to an indirect fixation (with covert attention) in order to evoke reliable SSVEPs in the EEG. And (2) whether oscillatory modulations in form of SSVEPs at the frequency stimulated at and the first harmonic response (cf. Ordikhani-Seyedlar et al., 2014) were present not only (a) when the flickering of the light stimulation was perceptible for the participant (i.e., presented at an intensity above a previously estimated individual perceptibility threshold) but also in (b) a less perceptible stimulation condition (i.e., during a stimulation with an intensity below the individual perceptibility threshold). Previous evidence suggests that SSVEPs are not strictly limited to local regions but rather engage broadly distributed functional networks. These studies suggest an increase in the coupling between the visual cortex and non-visual structures during the rhythmic stimulation (e.g., Lithari et al., 2016). However, it is still unclear in which stimulation protocols and under which circumstances the phenomenon of global entrainment occurs. Hence, we were interested in possible network-level processes evoked by the different rhythmic light stimulations. Therefore, we examined the hypothesis that a change in local cortical power has a large-scale influence by analyzing changes in the functional connectivity in the frequency stimulated at and its harmonic response as a response to the different rhythmic light stimulation conditions.

## 2. Methods

### 2.1. Participants

A total of 18 healthy participants (14 men) with a mean age of *M* = 27.33 years (*SD* = 3.99, range: 19–35 years) took part in this study and received a monetary reward for participation. All participants had normal or corrected-to-normal vision and a visual acuity above 0.7 measured via the Freiburg Visual Acuity Test (FrACT; Bach, 1996) by certified optometrists. Only participants reporting no neurological diseases (e.g., epilepsy), centrally effective medication, drug consumption, and psychiatric disorders were invited. All participants were informed about a potential (but rather small) risk of epileptic seizure due to the rhythmic visual stimulation (Vialatte et al., 2010). Prior to the experiment, we examined the individual perceptibility threshold. Participants signed a written informed consent according to the recommendations of the declaration of Helsinki. The study was approved by the ethics committee of the Medical Faculty of the University of Tuebingen, Germany. Four participants were excluded because they did not meet the inclusion criteria due to a high perceptibility threshold with an intensity higher than 9 mA, resulting in a total of 14 participants in the SSVEP-SNR analysis (11 men; age of *M* = 27.71 years, *SD* = 3.89). Further two participants were excluded in the functional connectivity analysis, resulting in a total of 12 participants (10 men; age of *M* = 26.83 years, *SD* = 3.80).

### 2.2. Apparatus and Stimuli

**Figure 1:**
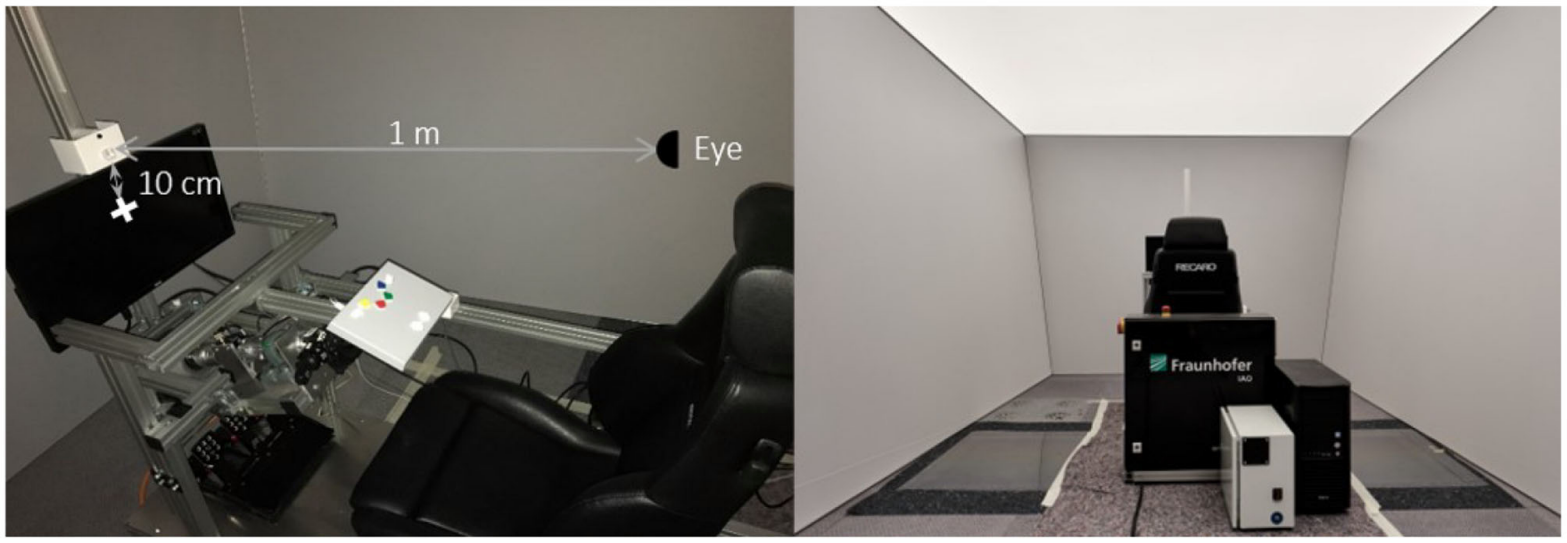
Experimental set-up. The experimental environment was a mock-up vehicle interior within a dark recording chamber. The light source was positioned with a distance of 1 m in front of the participant (left). Participants did not have to interact with the mock-up vehicle interior (e.g., the pedals or steering wheel) during the experiment.

**Figure 2:**
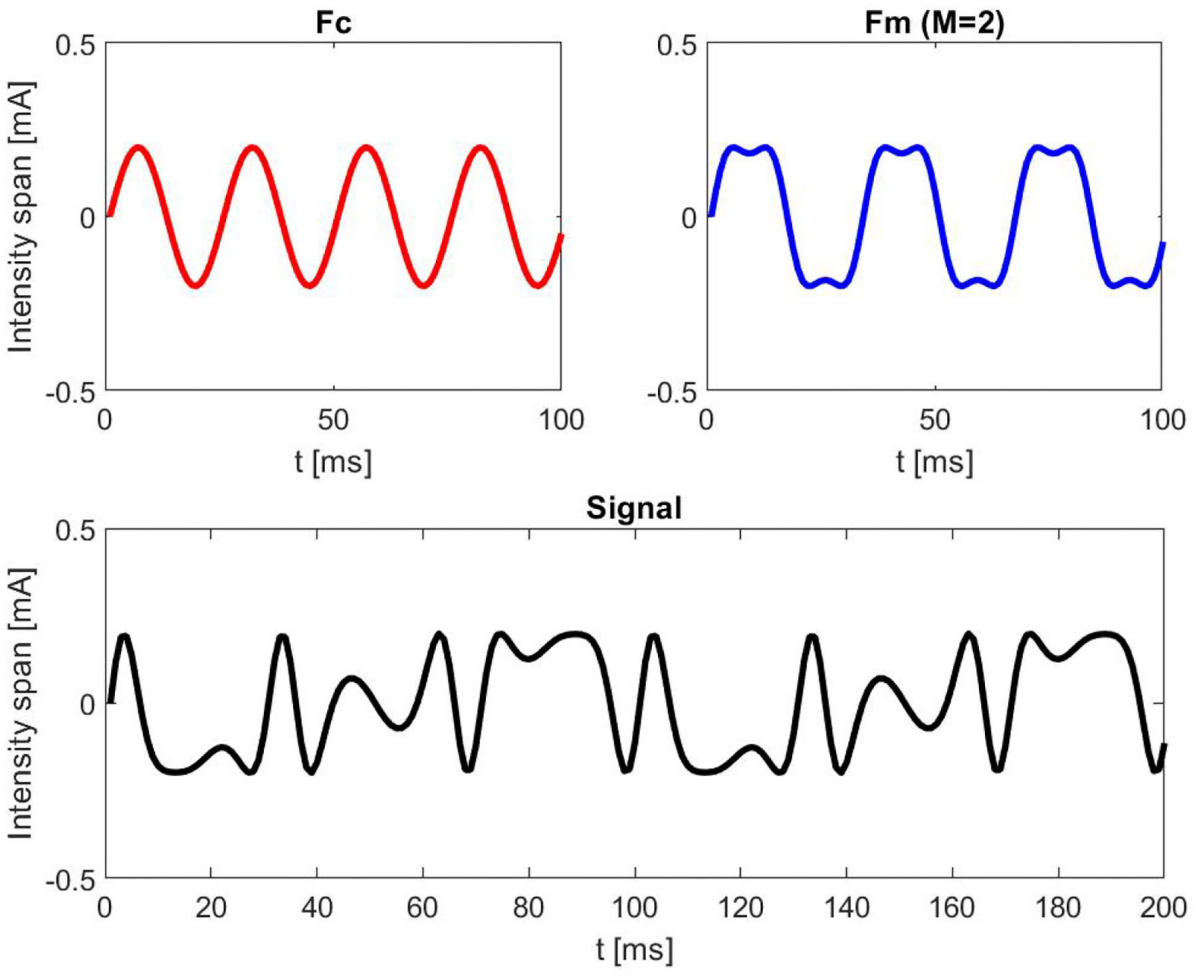
Illustrative simulation of a frequency modulated (FM) signal at 7 mA with a carrier frequency (*Fc*) of 40 Hz, modulation frequency (*Fm*) of 30 Hz, intensity span (*FCI*) of 0.2 mA, and a modulation index (*M*) of 2.

A warm white-colored light-emitting diode (2,800 K with a peak at 600 nm, diameter 0.5 cm, Model NSPL500DS, Nichia Corporation) was mounted at a distance of 1 m from the participants' nasion in front of a black computer screen. To illuminate a larger area of the retina and reduce glare, a homogeneous diffusor was placed in front of the LED, thereby covering 1.14° of the visual field. A cross-hair was positioned 10 cm below the LED as a fixation point in the covert condition, covering 5.7° of the visual field (see Figure 1). The distance between the light source and the cross-hair as the fixation point in the covert attention condition and between the participants and installation was chosen based on Dreyer et al. (2017). A close-to-real-time capable industrial PC (10 μs; 16-bit) was used to drive the LED at 10 Hz. For the synchronization of the stimulation and EEG recordings, we used a parallel port and CANopen interface. The stimuli were generated with a Beckhoff Framework programmed by AIOCAS S.a.r.l. using the following formula for FM stimulation protocols adapted from Dreyer and Herrmann (2015) and Dreyer et al. (2017):


signal=A+FCI*sin(2*π *Fc*t+(M*sin(2*π *Fm*t)))

where *A* represents the current intensity in mA at which the LED was driven and was estimated for each participant previously in a separate session in order to define the individual perceptibility threshold (IPT). *FCI* signifies the fluctuation of the current intensity span with 0.2 mA. *Fc* represents the carrier frequency (40 Hz) and *Fm* the corresponding modulation frequency (30 Hz). *M* is the modulation index (*M* = 2) and *t* is the time vector. The time course of the LED was tested with an oscilloscope (HAMEG hm 205). By choosing the *Fc* at 40 Hz and *Fm* at 30 Hz, we aimed to evoke SSVEP responses at 10 Hz (40–30 Hz). The carrier frequency was chosen based on Dreyer and Herrmann (2015), since the authors reported no significant differences in flicker perceptibility scores for carrier frequencies above 30 Hz. Figure 2 provides an exemplary simulation of FM signal at 7 mA.

### 2.3. Procedure

Participants were comfortably seated in a mock-up vehicle interior in a dark and quiet recording chamber (see Figure 1). Prior to the experiment, participants were informed about muscle and movement artifacts in EEG signals and instructed to keep movements at a minimum. The experiment started with a 3-min EEG baseline recording with eyes opened and fixated on a cross-hair under a shallowly illuminated static environmental light condition (i.e., not flickering with an average luminance at 20 cd/m). Afterwards, participants had to rate their sleep quality of the past night with the Leeds Sleep Evaluation Questionnaire (LSEQ; Parrott and Hindmarch, 1978) and momentary sleepiness with the Karolinska Sleepiness Scale (KSS; Akerstedt and Gillberg, 1990).

To investigate the role of perceptibility, we designed the rhythmic light stimulation either to be perceptibly flickering at an intensity above a previously estimated individual perceptibility threshold (i.e., IPT + 2 mA; A-IPT) or less perceptibly flickering at an intensity below the individual perceptibility threshold (i.e., IPT − 2 mA; B-IPT). The intensity was estimated prior to the main session in a separate pre-session for the overt and covert attention condition with the method of constant stimuli (Eisen-Enosh et al., 2017). In this pre-session, 14 stimuli each with 10 repetitions were presented separately for the overt and covert attention condition (total of 280 stimulation trials). Ten of the 14 stimuli were flickering (0.5, 1, 2, 3, 4, 5, 6, 7, 8, 9 mA) and four were static representing control stimuli (1, 3, 5, 7 mA). The attention condition was cued 8 s before stimulation onset with either an arrow (overt attention condition) or a cross-hair (covert attention condition) signaling the participant to either fixate on the LED (overt attention condition) above or the cross-hair (covert attention condition) during the stimulation. Afterwards, the stimulation was presented for at least 8 s up to a maximum of 30 s. In the pre-session, participants were asked to decide whether they perceive the stimulation as flickering or not by pressing a button as soon as possible but within the 30 s stimulation window. Each stimulation trial was followed by an inter-stimulus interval (ISI) of 10 s. The IPT was defined as the current intensity at which the flicker stimuli were not detected in 50% of the presentations (Eisen-Enosh et al., 2017). For this purpose, a sigmoid function was determined for each participant.

**Figure 3:**
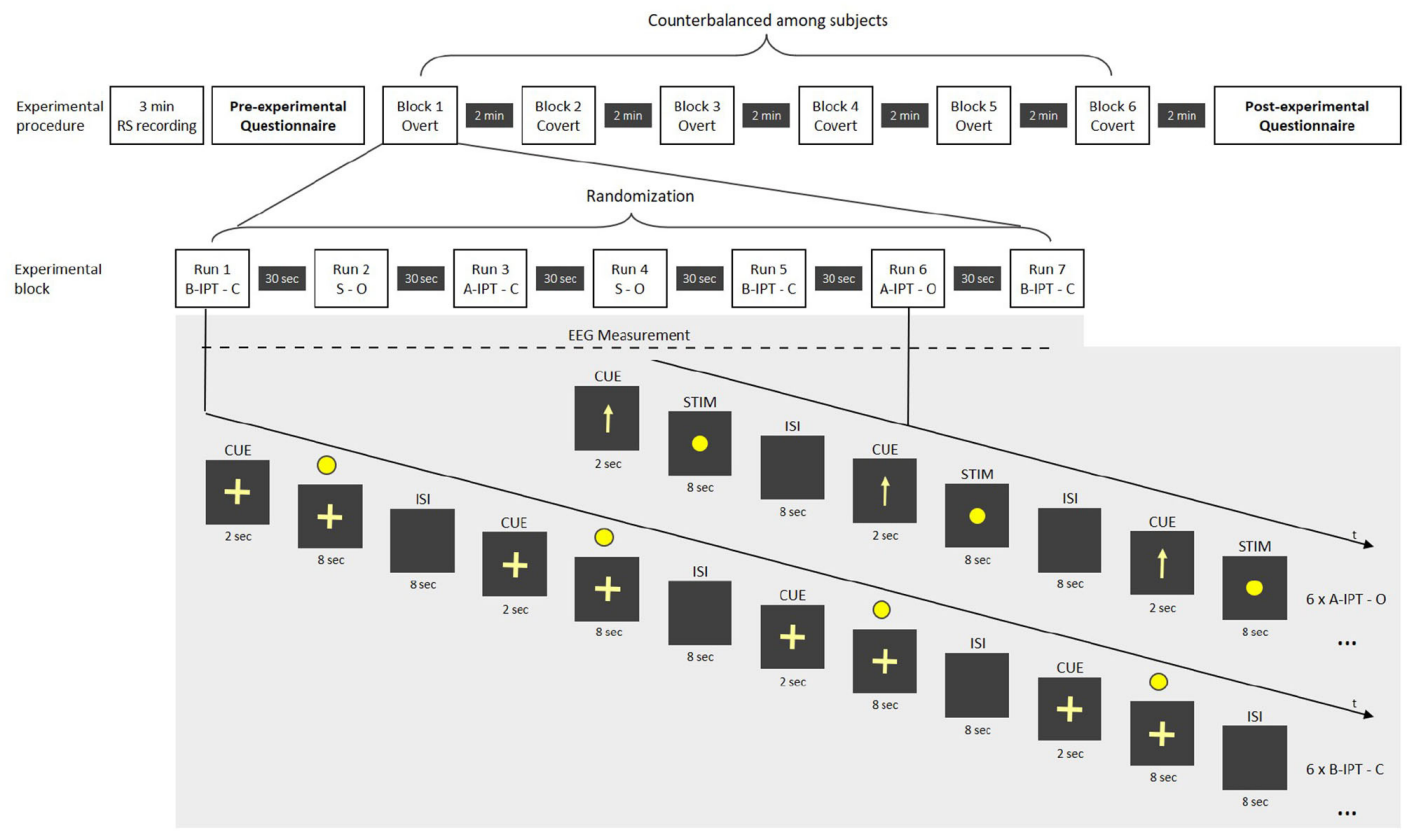
Overview of the experimental procedure in the main session. RS, resting state; STIM, stimulation; CUE, cue before the stimulation signaling the attention-based condition; ISI, inter-stimulus interval; S, static stimulation; A-IPT, above the individual threshold stimulation; B-IPT, below the individual threshold stimulation; C, covert attention condition; O, overt attention condition.

In the main session, a third static condition without any flickering was used as a manipulation check for the subjective perceptibility ratings. Stimuli were presented in 2 (attention; overt − covert) ×3 (perceptibility; A-IPT − B-IPT − static) conditions with 42 repetitions per stimuli and a total amount of 252 stimuli. All participants attended all conditions. Figure 3 provides an overview of the experimental procedure of the main session. In the overt attention condition, participants had to fixate directly on the LED during the stimulation. In the covert attention condition, they were asked to fixate on a cross-hair below the LED. During the EEG set-up, participants' eyes could adjust to the dim light conditions. In the main session, the conditions were presented block-wise with seven randomized runs within each block. The attention condition was cued 2 s with either an arrow (overt) or cross-hair (covert) before stimulation onset. The following stimulation length was determined at 8 s in the main session (compared to the pre-session where the stimulation lasted up to 30 s but participants could stop the stimulation after 8 s by button press). Each run comprised of a series of six repetitions of the same stimulation condition with an ISI of 8 s. The order of the conditions within a block was Latin-square counterbalanced with the constraint that the attention conditions (overt and covert) (a) are alternated among runs and blocks and (b) are equally balanced and pseudo-randomized among participants regarding their order and starting stimulation condition. In total, each run lasted 108 s with a break of 30 s afterwards. Each block was 966 s long and followed by a 2-min break. After each run, participants had to rate their sleepiness on the 9-point KKS extended with a visual scale and subjective flickering perceptibility of the stimulation on a 5-point visual analog scale (adapted from Dreyer and Herrmann, 2015). After each block, we additionally asked them to provide ratings on a visual comfort scale (Steinemann et al., 2019) and concerning their subjective well-being (Birchler-Pedross et al., 2009). In a post-experimental questionnaire, they were asked about potential confounding factors (e.g., noise level, air quality, temperature; Eklund and Boyce, 1996).

### 2.4. EEG Data Acquisition

The scalp EEG potentials were recorded (BrainAmp, Brainproducts GmbH, Germany) from 32 positions according to the international 10–20 system with Ag/AgCl electrodes (actiCAP, Brainproducts GmbH, Germany). The left mastoid was used as common reference and EEG was grounded to FCz. The impedance was kept below 20 k℧ at the onset of each session. EEG data were digitized at 1 kHz, high-pass filtered with a time constant of 10 s, and stored for off-line analysis using the BrainVision Recorder Software (Brain Products GmbH, Munich, Germany).

### 2.5. Data Analysis

The data analysis was performed with custom written or adapted scripts in MATLAB^®^ and python™.

#### 2.5.1. EEG Pre-processing

First, the continuous EEG signals were de-trended, zero-padded, and re-referenced to mathematically linked mastoids using the average of the two mastoid sensors (TP9 and TP10; Nunez and Srinivasan, 2006). Artifact afflicted EEG channels, as determined by visual inspection, were removed. Hence, we excluded five EEG channels (T7, T8, PO9, PO10, and CP1) from further analysis to maintain the same number of channels in each participant. The EEG signals were band-pass filtered between 0.5 and 42 Hz using a first order zero-phase lag Finite Impulsive Response (FIR) filter. Next, EEG signals were split into segments of different sizes: For the SNR analysis, we used segments from 1,000 to 8,000 ms after stimulation onset. For the analysis of the functional connectivity, we were interested in characterizing resources demanded by the visual cortex when recruiting anterior cortical regions in response to rhythmic light stimulation (Lithari et al., 2016). Therefore, we chose segments from 2,000 to 6,000 ms for the analysis of functional connectivity to exclude possible effects rather explained by the pure effect of stimulation on- and offset than local power entrainment. EEG segments were rejected when containing a maximum deviation above 200 μV in any of the frontal EEG channels (Fp1, Fp2). Next, we performed an independent component analysis (ICA) separately for the conditions using the logistic infomax ICA algorithm as implemented in the EEGlab toolbox (Delorme and Makeig, 2004) to remove ocular movement, cardiac, and muscular artifacts. This was done by careful visual inspection of the topography, times course, and power spectral intensity of the ICA components (Chaumon et al., 2015; Hipp and Siegel, 2013). On average *M* = 8.29 ± 3.67 (B-IPT, overt attention), *M* = 7.58 ± 3.75 (A-IPT, overt attention), *M* = 8.0 ± 3.72 (B-IPT, covert attention), and *M* = 8.50 ± 4.34 (A-IPT, covert attention) components were rejected.

#### 2.5.2. Detecting Significant SSVEPs by Analyzing the SNR

To detect SSVEPs and perform the spectral analysis, the 7,000 ms segments from the occipital channels (O1, Oz, O2) were cut in 1,000 ms epochs with a sliding window of 100 ms overlap, separately for the conditions, as suggested by Dreyer and Herrmann (2015). The 1,000 ms epochs were zero-mean corrected by their respective mean values. The SSVEPs were calculated by averaging the epochs as well as occipital channels to an ERP for each condition. The ERP was then decomposed into its frequency components using the discrete fast Fourier transform (FFT) with a frequency resolution of 1 Hz. To detect significant SSVEPs, we proposed two approaches: In the first approach, we calculated the SNR by dividing the 10 Hz SSVEP amplitudes of the overlapping evoked spectra by the average amplitude of the two neighboring frequencies (9 and 11 Hz) according to Meigen and Bach (1999).


$$s\;value=\frac{SSVEP\;signal\;magnitude}{\frac{noise\;magnitude\;at\;the\;lower\;neighbor-at\;the\;upper\;neighbor}{2}}$$

In the second approach, as suggested by Norcia et al. (2015) and Rossion et al. (2012), we calculated the SNR by dividing the 10 Hz SSVEP amplitudes by the average amplitude of the five neighboring frequencies at each side (5–9 and 11–15 Hz).

We used the SNR thresholds suggested by Meigen and Bach (1999) with significance levels for SNRs at s values of 2.82, *p* < 0.05, 4.55, *p* < 0.01, and 8.40, *p* < 0.001. We further z-scored the SSVEP power using the mean amplitude of the five surrounding frequency bins divided by the standard deviation of the five surrounding bins allowing to compare the z-scored SSVEP power to the threshold of significance with a z-score of 1.96 corresponding to *p* < 0.05, 2.58 to *p* < 0.01, and 3.29 to *p* < 0.001 (Liu-Shuang et al., 2014).

#### 2.5.3. Estimation of Functional Connectivity

To study global oscillatory effects (see Lithari et al., 2016), we further analyzed functional cortical networks. For the calculation of functional connectivity (FC), we used the imaginary part of the coherence function (iCOH; Nolte et al., 2004). The iCOH function disregards relations at zero (or close to zero) time lag and is, thus, less sensitive to volume conduction properties when functional connectivity is studied at the sensor level. It indicates the relative coupling of phases, i.e., the time-lag between two brain processes. Since the original iCOH function might exhibit a spatial bias toward long-range synchronizations, we used the corrected version of the iCOH function (ciCOH) as suggested by Ewald et al. (2012). ciCOH shares the same properties as the originally proposed version but includes additional features to compensate for the preference of remote interactions. The estimation of the ciCOH is based on the complex coherency function. Hence, from each valid segment of 4,000 ms, cross-spectral densities were calculated using an FFT of the EEG time series and spectrally smoothing the data according to a multi-tapering approach as implemented in the Chronux toolbox (Mitra and Bokil, 2007; Chronux, 2016). Here, a 1,000 ms window size with a step size of 100 ms was used. While the 1,000 ms segments were tapered using slepian functions, the number of tapers applied was defined according to the equation 2 * bandwidth − 1, (Percival and Walden, 1993), inserting a bandwidth of 3 and resulting in five tapers for spectral smoothing with a spectral bandwidth of δ*f* = 0.98 Hz. From the cross-spectra, the complex coherency function between channel pairs was defined as the normalized cross-spectrum for channels *i* and *j*:


$$COH_{ij}\left(f\right)=\frac{s_{ij}\left(f\right)}{\sqrt{s_{ii}\left(f\right)s_{jj}\left(f\right)}}$$

where *S*_*ij*_(·) is the cross-spectrum between channels *i* and *j*, and *S*_*ii*_(·), *S*_*jj*_(·) represented the auto-spectra for channels *i* and *j*, respectively. From the complex coherency function, the ciCOH was defined (Ewald et al., 2012):


$$ciCOH_{ij}\left(f\right)=\frac{Im\left(COH_{ij}\left(f\right)\right)}{\sqrt{1-Re{\left(COH_{ij}\right)}^{2}}}$$

where *lm*(·) and *Re*(·) denote the imaginary and real part, respectively. The ciCOH was fisher z-transformed to fit a Gaussian distribution (Rosenberg et al., 1989; Nolte et al., 2004). We evaluated the FC within the pre-defined frequencies of interest (FOI): alpha band (8–12 Hz) and first harmonic beta band (18–22 Hz). Since potential differences between the stimulations might be limited to the exact stimulation frequency, we further investigated the functional connectivity at the alpha (10 Hz) and beta peak (20 Hz). We systematically evaluated the FC networks between the visual cortex (VIC) and electrodes over frontal(-central) regions (Fp1, Fp2, AFz, F7, F3, Fz, F4, F8, FC5, FC1, FC2, FC6), for each FOI, by defining O1, Oz, and O2 (electrodes over VIC) as the seed electrodes. Henceforth, the functional connectivity is referred as occipito-frontal(-central) functional network. In a next step, the FC measure was obtained by calculating the absolute value of the ciCOH during the stimulation:


$$ciCOH_{Seed,j}\left(FOI\right)=abs{\left(ciCOH_{Seed,j}\left(FOI\right)\right)}_{TOI}$$

where *Seed* denotes the seed electrode and *abs* indicates the absolute value of ciCOH.

#### 2.5.4. Statistical Analysis of the Subjective Ratings, SNR, and Functional Connectivity

For the inferential statistic, we used non-parametric Friedman χ^2^ tests to account for outliers and a non-normal distribution of the variables. To examine the differences between two conditions, non-parametric *post-hoc* Wilcoxon signed rank tests were conducted using the false discovery rate (FDR) with the Benjamini–Hochberg method as multiple comparison correction.

## 3. Results

### 3.1. Individual Perceptibility Threshold

**Figure 4:**
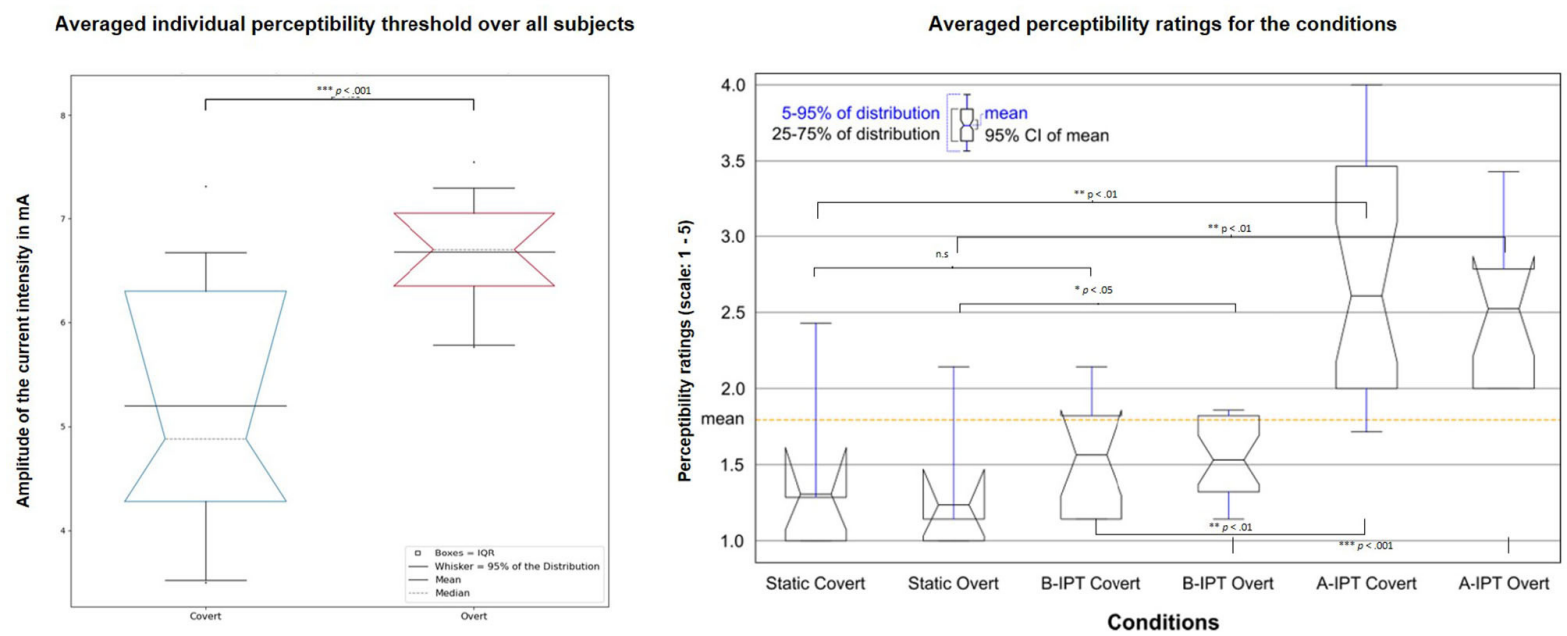
Comparison of the average individual perceptibility threshold for the overt and covert attention condition (left) and perceptibility ratings among the six stimulation conditions (right). (Left) The average individual perceptibility threshold (IPT) using the current intensity examined in the pre-session for the covert (left box) and overt (right box) attention condition. Stimuli were designed to have an intensity above the individual perceptibility threshold (A-IPT) with 2 mA over the IPT (IPT + 2 mA) and below the individual perceptibility threshold (B-IPT) with 2 mA below the IPT (IPT − 2 mA). The gray dashed line represents the bootstrapped median (2,000 repetitions) and notches of the boxes its 95% confidence interval (CI). (Right) Comparison of the perceptibility ratings on a 5-point scale (adapted from Dreyer and Herrmann, 2015) among the six stimulation conditions. The orange dashed line represents the average mean over all conditions. A-IPT, above the individual threshold stimulation; B-IPT, below the individual threshold stimulation.

The intensity of the individual 50% flickering thresholds (i.e., the intensity at which 50% of the stimuli were rated as flickering) differed significantly between the two attention conditions with *p* ≤ 0.001 and an average intensity of *M* = 6.47 (*SD* = 0.96) mA in the overt and *M* = 5.10 (*SD* = 1.27) mA in the covert attention condition. Hence, the average intensity in overt attention condition was *M* = 8.47 (IPT + 2) mA for the B-IPT and *M* = 4.47 (IPT − 2) mA for the A-IPT stimulation. In the covert attention condition, the average current intensity was *M* = 7.06 (IPT + 2) mA for the B-IPT and *M* = 3.17 (IPT − 2) mA for the A-IPT stimulation (see Figure 4, left).

### 3.2. Subjective Ratings

Participants reported similar sleep conditions, sleep quality, and amount of sleep. Results of the KSS revealed no differences between the conditions regarding fatigue or sleepiness. Regarding the subjective perceptibility ratings, significant differences in the perceived flickering of the stimulation among conditions show a subjectively successful manipulation, $\chi_{\left(5\right)}^{2}\;=\;38.445,p\;\leq \;0.001.$ *Post-hoc* multiple comparison corrected tests revealed a significant difference between the static condition and stimulation above the individual perceptibility threshold (A-IPT) with higher ratings for A-IPT not only in the overt condition, *p*_*corrected*_ = 0.008, but also in the covert condition, *p*_*corrected*_ = 0.002. Furthermore, the A-IPT stimulation was significantly more perceptible than the B-IPT stimulation in the overt, *p*_*corrected*_ ≤ 0.001, and covert condition, *p*_*corrected*_ = 0.006. While participants rated more flickering in the B-IPT stimulation condition compared to the static condition during overt attention, *p*_*corrected*_ = 0.042, no difference was reported in the covert condition, *p*_*corrected*_ = 0.116 (see Figure 4, right). No difference between the blocks could be observed for the visual comfort scale in the dimensions (1) liking, $\chi_{\left(5\right)}^{2}=8.265,p=0.142$, (2) brightness, $\chi_{\left(5\right)}^{2}=10.197,p=0.070$, (3) light color, $\chi_{\left(5\right)}^{2}=3.125,p=0.681$, (4) blinding, $\chi_{\left(5\right)}^{2}=5.241,p=0.387$, (5) vigilance, $\chi_{\left(5\right)}^{2}=9.123,p=0.104$, (6) concentration, $\chi_{\left(5\right)}^{2}=4.280,p=0.510$, and (7) fixation, $\chi_{\left(5\right)}^{2}=8.920,p=0.112$. There were significant differences between blocks regarding the dimensions of (8) effort to stay focused, $\chi_{\left(5\right)}^{2}=13.710,p=0.018$, and (9) sleepiness, $\chi_{\left(5\right)}^{2}=17.514,p=0.004$. However, after FDR correction, no *post-hoc* comparison was significant. There were no differences among blocks for the subjective well-being ratings in the dimensions mood, motivation, subjective physical well-being, satisfaction, and tension. However, participants rated the dimensions concentration, $\chi_{\left(5\right)}^{2}=36.423,p\leq 0.001$, effort, $\chi_{\left(5\right)}^{2}=34.230,p\leq 0.001$, sleepiness, $\chi_{\left(5\right)}^{2}=20.387,p\leq 0.001$, tiredness, $\chi_{\left(5\right)}^{2}=28.436,p\leq 0.001$, and fatigue, $\chi_{\left(5\right)}^{2}=25.058,p\leq 0.001$, different among blocks with decreasing concentration and increasing effort, sleepiness, and fatigue over time. No confounding environmental effects (noise level, air quality, etc.) were reported in the post-experimental questionnaire.

### 3.3. EEG SNR Results

**Figure 5:**
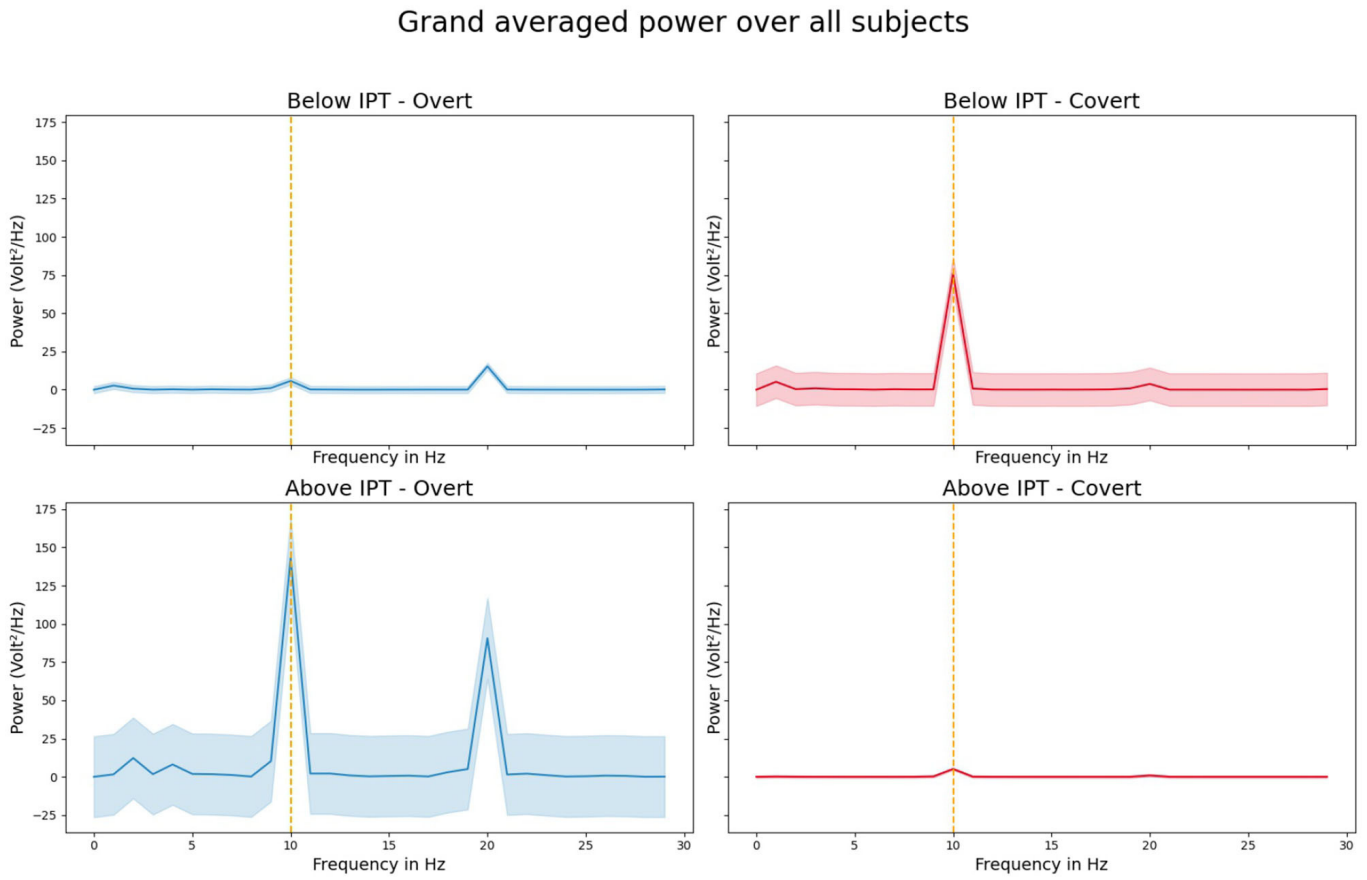
Grand averaged steady-state visual evoked potential (SSVEP) power over all participants during the 10 Hz stimulation for the conditions below (upper row; B-IPT) and above (lower row; A-IPT) the individual perceptibility threshold in the overt (left) and covert (right) attention condition. Shaded area represents the standard deviation.

We were able to evoke 10 Hz SSVEPs in all except one participant and in all four conditions. Table 1 provides the (1) SNR values and (2) z-scored SSVEP power for the comparison with the (a) neighboring frequencies 9 and 11 Hz and (b) surrounding five neighboring frequencies 5–9 and 11–15 Hz for all participants for the B-IPT and A-IPT stimulation in the overt attention condition (upper part) and covert attention condition (lower part). Using the SSVEP detection method suggested by Meigen and Bach (1999), all SNR values for the A-IPT stimulation in the covert and overt attention condition and for the B-IPT stimulation in the overt attention condition were above the suggested threshold of *s* = 2.82, *p* < 0.05, and mostly even above *s* = 8.40, *p* < 0.001 (Meigen and Bach, 1999). For the B-IPT stimulation in the covert attention condition, only the SNR calculated via the directly neighboring frequencies 9 and 11 Hz of participant 14 could not reach the significance threshold. When comparing the z-scored SSVEP power for the direct neighboring and surrounding five frequencies as suggested by Rossion et al. (2012) and Liu-Shuang et al. (2014), values of all conditions and participants were above the significance threshold of 1.96, *p* < 0.05, and even above a z-score of 3.29 , *p* < 0.001. We investigated possible differences in the SNR values and SSVEP amplitudes at 10 Hz of the conditions. The four conditions differed neither in the SNR values calculated via the directly neighboring frequencies 9 and 10 Hz, $\chi_{\left(3\right)}^{2}\;=\;-0.236,p\;=\;0.817$, nor in those calculated via the five surrounding frequencies, $\chi_{\left(3\right)}^{2}\;=\;1.486,p\;=\;0.163$, nor in the SSVEP amplitudes at 10 Hz, $\chi_{\left(3\right)}^{2}\;=\;0.983,p\;=\;0.344$. Hence, no difference between the conditions could be found regarding their overlapping evoked power spectra and SNR significance values. Figure 5 shows the power averaged over all participants for the stimulation above (A-IPT) and below (B-IPT) the individual perceptibility threshold during overt and covert attention, respectively.

**Table 1:** Signal-to-noise ratio (SNR) values and z-scored steady-state visual evoked potential (SSVEP) power at 10 Hz for each participant and condition (upper: overt, lower: covert) with SSVEP amplitude values in *Volt*^2^/*Hz* in brackets after the directly neighboring frequencies.

	**B-IPT Overt**				**A-IPT Overt**			
**Neighboring frequencies**	**9 and 11 Hz**		**5–9 and 11–15 Hz**		**9 and 11 Hz**		**5–9 and 11–15 Hz**	
**Participants**	**SNR (power)**	**z-scored SSVEP power (*Mean*_*nfreqs*_ ± *SD*_*nfreqs*_)**	**SNR (power)**	**z-scored SSVEP power (*Mean*_*nfreqs*_ ± *SD*_*nfreqs*_)**	**SNR (power)**	**z-scored SSVEP power (*Mean*_*nfreqs*_ ± *SD*_*nfreqs*_)**	**SNR (power)**	**z-scored SSVEP power (*Mean*_*nfreqs*_ ± *SD*_*nfreqs*_)**
1	74.02^***^(1.01)	98.42^***^(0.01 ± 0.01)	249.87^***^(1.01)	148.43^***^(0.00 ± 0.01)	22.56^***^(2.32)	410.8^***^(0.10 ± 0.01)	78.82^***^(2.32)	61.18^***^(0.03 ± 0.04)
2	46.96^***^(0.58)	71.20^***^(0.01 ± 0.01)	153.83^***^(0.58)	100.52^***^(0.00 ± 0.01)	5.88^**^(0.07)	6.07^***^(0.01 ± 0.01)	17.41^***^(0.07)	11.05^***^(0.00 ± 0.01)
3	4.31^*^(30.88)	4.14^***^(7.16 ± 5.73)	13.39^***^(30.88)	7.74^***^(2.31 ± 3.69)	226.20^***^(3.52)	366.09^***^(0.02 ± 0.01)	836.42^***^(3.52)	482.06^***^(0.00 ± 0.01)
4	1994.70^***^(6.73)	5100.09^***^(0.00 ± 0.00)	4454.97^***^(6.73)	4272.47^***^(0.00 ± 0.00)	14.38^***^(0.38)	78.87^***^(0.03 ± 0.00)	58.36^***^(0.38)	36.63^***^(0.01 ± 0.01)
5	75.05^***^(2.70)	111.48^***^(0.04 ± 0.02)	220.48^***^(2.70)	165.89^***^(0.01 ± 0.02)	44.38^***^(1.32)	325.96^***^(0.03 ± 0.00)	196.57^***^(1.32)	112.62^***^(0.01 ± 0.01)
6	168.79^***^(2.95)	1100.07^***^(0.00 ± 0.00)	413.99^***^(2.95)	353.96^***^(0.01 ± 0.01)	66.68^***^(1.46)	125.24^***^(0.02 ± 0.01)	269.17^***^(1.46)	149.27^***^(0.01 ± 0.01)
7	10.94^***^(0.42)	25.72^***^(0.04 ± 0.01)	36.69^***^(0.42)	26.77^***^(0.01 ± 0.02)	8.77^***^(3.28)	48.03^***^(0.37 ± 0.06)	30.68^***^(3.28)	22.57^***^(0.11 ± 0.14)
8	25.41^***^(3.61)	61.83^***^(0.14 ± 0.06)	68.91^***^(3.61)	65.06^***^(0.05 ± 0.05)	14.50^***^(3.14)	32.10^***^(0.22 ± 0.09)	48.14^***^(3.14)	34.57^***^(0.07 ± 0.09)
9	146.23^***^(2.97)	217.60^***^(0.02 ± 0.01)	421.18^***^(2.97)	314.27^***^(0.01 ± 0.01)	328.06^***^(1.32)	615.58^***^(0.00 ± 0.00)	581.76^***^(1.32)	661.77^***^(0.00 ± 0.00)
10	19.36^***^(0.10)	39.26^***^(0.01 ± 0.00)	53.45^***^(0.10)	45.84^***^(0.00 ± 0.00)	69.34^***^(1.42)	158.2^***^(0.02 ± 0.01)	279.47^***^(1.42)	161.68^***^(0.01 ± 0.01)
11	163.49^***^(0.96)	302.23^***^(0.01 ± 0.00)	600.48^***^(0.96)	367.71^***^(0.00 ± 0.00)	759.07^***^(3.06)	1274.76^***^(0.00 ± 0.00)	2094.19^***^(3.06)	1724.33^***^(0.00 ± 0.00)
12	142.93^***^(11.24)	348.37^***^(0.08 ± 0.03)	556.91^***^(11.24)	340.74^***^(0.02 ± 0.03)	53.43^***^(3.06)	71.54^***^(0.06 ± 0.04)	169.60^***^(3.06)	110.54^***^(0.02 ± 0.03)
13	7.45^**^(10.18)	8.13^***^(1.37 ± 1.08)	32.33^***^(10.18)	13.79^***^(0.31 ± 0.72)	23.00^***^(1965.25)	33.04^***^(85.41 ± 56.89)	67.22^***^(1965.25)	49.74^***^(29.23 ± 38.92)
14	49.13^***^(6.24)	72.90^***^(0.13 ± 0.08)	210.03^***^(6.24)	101.00^***^(0.03 ± 0.06)	400.15^***^(15.30)	2281.55^***^(0.04 ± 0.01)	1004.64^***^(15.3)	1008.09^***^(0.02 ± 0.02)
*Mean*	209.20^***^ (5.76)	540.10^***^ (0.65 ± 0.50)	534.75^***^ (5.76)	451.73^***^ (0.20 ± 0.33)	145.46^***^ (143.21)	416.28^***^ (6.17 ± 4.08)	409.47^***^ (143.21)	330.44^***^ (2.11 ± 2.80)
*SD*	498.58 (7.77)	1293.83 (1.84 ± 1.48)	1103.62 (7.77)	1067.36 (0.59 ± 0.95)	209.51 (505.36)	612.31 (21.98 ± 14.65)	555.58 (505.36)	479.13 (7.51 ± 10.01)
	**B-IPT Covert**				**A-IPT Covert**			
**Neighboring frequencies**	**9 and 11 Hz**		**5–9 and 11–15 Hz**		**9 and 11 Hz**		**5–9 and 11–15 Hz**	
**Participants**	**SNR (power)**	**z-scored SSVEP power (*Mean*_*nfreqs*_ ± *SD*_*nfreqs*_)**	**SNR (power)**	**z-scored SSVEP power (*Mean*_*nfreqs*_ ± *SD*_*nfreqs*_)**	**SNR (power)**	**z-scored SSVEP power (*Mean*_*nfreqs*_ ± *SD*_*nfreqs*_)**	**SNR (power)**	**z-scored SSVEP power (*Mean*_*nfreqs*_ ± *SD*_*nfreqs*_)**
1	43.41^***^(1.89)	65.48^***^(0.04 ± 0.03)	167.91^***^(1.89)	91.18^***^(0.01 ± 0.02)	265.51^***^(2.69)	415.5^***^(0.01 ± 0.01)	886.6^***^(2.69)	577.14^***^(0.00 ± 0.00)
2	59.11^***^(0.35)	70.49^***^(0.01 ± 0.00)	134.61^***^(0.35)	114.98^***^(0.00 ± 0.00)	183.13^***^(0.55)	187.62^***^(0.00 ± 0.00)	624.51^***^(0.55)	322.27^***^(0.00 ± 0.00)
3	163.14^***^(933.8)	223.59^***^(5.72 ± 4.15)	343.03^***^(933.8)	347.35^***^(2.72 ± 2.68)	15.35^***^(3.28)	16.23^***^(0.21 ± 0.19)	69.28^***^(3.28)	27.27^***^(0.05 ± 0.12)
4	67.65^***^(2.30)	347.83^***^(0.03 ± 0.01)	301.13^***^(2.30)	169.41^***^(0.01 ± 0.01)	346.22^***^(2.29)	699.58^***^(0.01 ± 0.00)	778.75^***^(2.29)	821.11^***^(0.00 ± 0.00)
5	99.82^***^(3.83)	1881.82^***^(0.04 ± 0.00)	294.79^***^(3.83)	263.59^***^(0.01 ± 0.01)	26.3^***^(0.86)	81.37^***^(0.03 ± 0.01)	122.54^***^(0.86)	62.6^***^(0.01 ± 0.01)
6	58.5^***^(1.11)	312.67^***^(0.02 ± 0.00)	183.02^***^(1.11)	156.95^***^(0.01 ± 0.01)	33.26^***^(0.88)	828.79^***^(0.03 ± 0.00)	134.83^***^(0.88)	86.35^***^(0.01 ± 0.01)
7	1005.82^***^(39.76)	1453.06^***^(0.04 ± 0.03)	1580.19^***^(39.76)	1609.32^***^(0.03 ± 0.02)	5.82^**^(0.52)	681.39^***^(0.09 ± 0.00)	15.04^***^(0.52)	15.71^***^(0.03 ± 0.03)
8	32.78^***^(13.24)	37.94^***^(0.40 ± 0.34)	142.83^***^(13.24)	60.52^***^(0.09 ± 0.22)	205.17^***^(10.35)	776.47^***^(0.05 ± 0.01)	793.11^***^(10.35)	514.84^***^(0.01 ± 0.02)
9	111.00^***^(0.87)	250.69^***^(0.01 ± 0.00)	364.25^***^(0.87)	272.06^***^(0.00 ± 0.00)	266.35^***^(4.52)	518.47^***^(0.02 ± 0.01)	660.17^***^(4.52)	581.38^***^(0.01 ± 0.01)
10	5.97^**^(0.25)	10.42^***^(0.04 ± 0.02)	18.23^***^(0.25)	13.69^***^(0.01 ± 0.02)	7.08^**^(0.28)	16.26^***^(0.04 ± 0.02)	27.47^***^(0.28)	16.43^***^(0.01 ± 0.02)
11	30.82^***^(0.21)	106.44^***^(0.01 ± 0.00)	70.57^***^(0.21)	83.31^***^(0.00 ± 0.00)	67.91^***^(0.17)	109.24^***^(0.00 ± 0.00)	139.15^***^(0.17)	152.98^***^(0.00 ± 0.00)
12	723.45^***^(55.79)	1615.21^***^(0.08 ± 0.03)	2846.91^***^(55.79)	1699.64^***^(0.02 ± 0.03)	125.58^***^(17.97)	441.69^***^(0.14 ± 0.04)	408.41^***^(17.97)	316.69^***^(0.04 ± 0.06)
13	50.89^***^(9.50)	80.44^***^(0.19 ± 0.12)	134.33^***^(9.50)	110.62^***^(0.07 ± 0.09)	7.09^**^(11.13)	11.95^***^(1.57 ± 0.80)	32.31^***^(11.13)	15.2^***^(0.34 ± 0.71)
14	1.60 (0.16)	3.52^***^(0.10 ± 0.02)	5.25^**^(0.16)	3.55^***^(0.03 ± 0.04)	211.82^***^(16.28)	355.12^***^(0.08 ± 0.05)	817.06^***^(16.28)	456.67^***^(0.02 ± 0.04)
*Mean*	175.29^***^ (75.93)	461.40^***^ (0.48 ± 0.34)	470.51^***^ (75.93)	356.88^***^ (0.22 ± 0.23)	126.19^***^ (5.13)	367.12^***^ (0.16 ± 0.08)	393.52^***^ (5.13)	283.34^***^ (0.04 ± 0.07)
*SD*	289.32 (238.48)	634.78 (1.46 ± 1.06)	758.48 (238.48)	538.37 (0.70 ± 0.68)	113.84 (5.96)	289.54 (0.395 ± 0.20)	335.22 (5.96)	258.49 (0.09 ± 0.18)

A-IPT, above the individual threshold stimulation; B-IPT, below the individual threshold stimulation. Mean and standard deviation of the neighboring frequencies used for the z-score calculation are provided in brackets after the z-scored SSVEP power. Significance thresholds for the (1) SNRs (Meigen and Bach, 1999): 2.82 for a ^*^p < 0.05, 4.55 for a ^**^p < 0.01, and 8.40 for a ^***^p < 0.001, and (2) z-scored SSVEP power: 1.96 for a ^*^p <0.05, 2.58 for a ^**^p < 0.01, and 3.29 for a ^***^p < 0.001.

### 3.4. EEG Functional Connectivity Results

**Figure 6:**
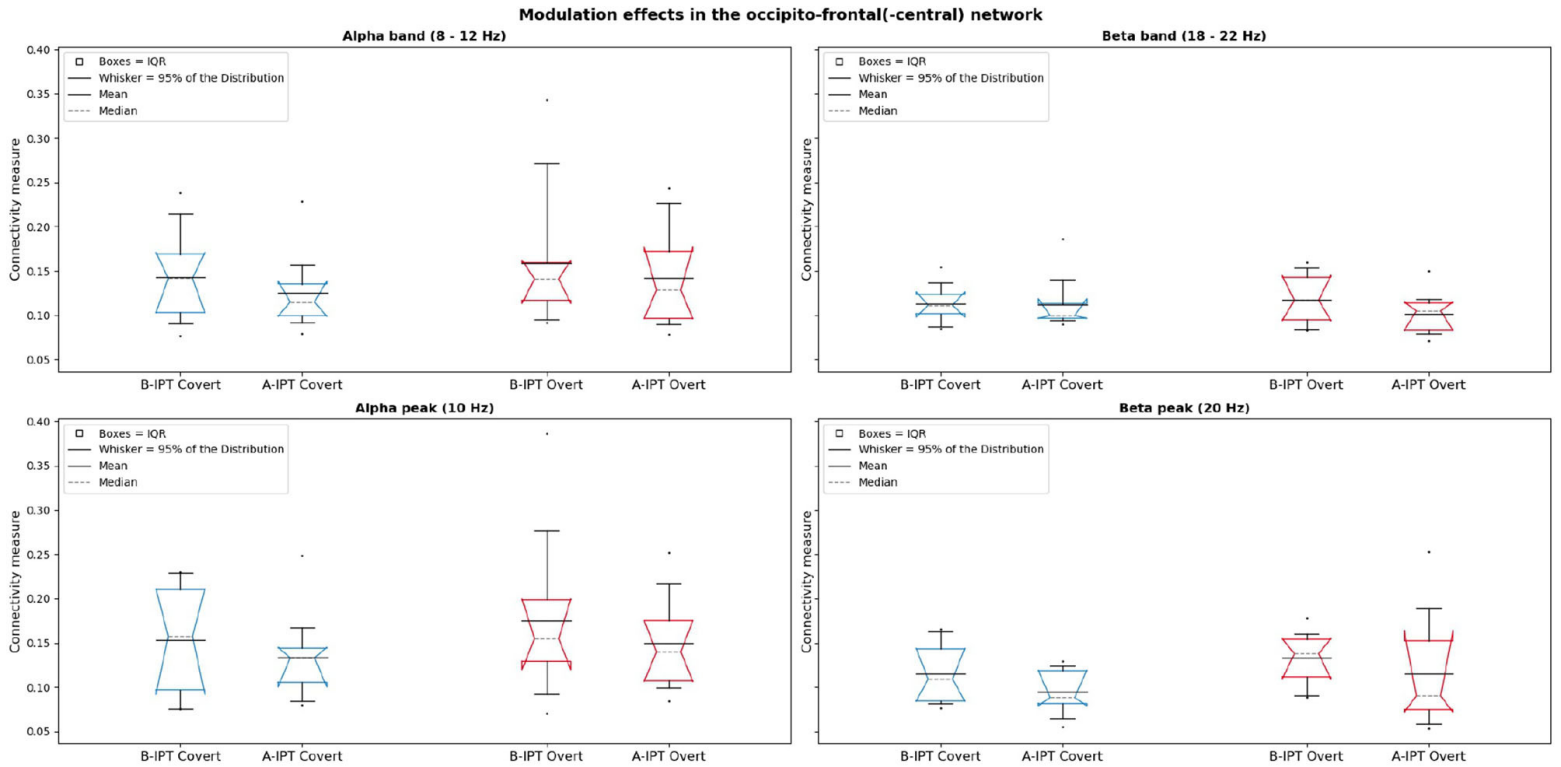
Comparison of the conditions regarding the connectivity measures in the occipito-frontal(-central) network in alpha (left) and the first harmonic response beta (right) for the peak (10 and 20 Hz) and band (8–12 and 18–22 Hz). The gray dashed lines represent the bootstrapped medians (2,000 repetitions) and the notches of the boxes the 95% confidence interval (CI) of the median.

Results of the functional connectivity analysis revealed no significant difference between the conditions in the alpha band, $\chi_{\left(3\right)}^{2}=-1.348,p=0.205$, and beta band, $\chi_{\left(3\right)}^{2}=1.711,p=0.115$, as well as alpha peak, $\chi_{\left(3\right)}^{2}=-1.288,p=0.224$, and beta peak, $\chi_{\left(3\right)}^{2}=-0.931,p=0.372$ in the occipito-frontal(-central) network. Figure 6 shows the connectivity in the alpha and beta band as well as in the alpha and beta peaks for all conditions with the mean, median, and the 95% CI of the median (notches of the boxes).

## 4. Discussion

Within our study, we investigated effects of attention and perceptibility during frequency-modulated rhythmic light stimulations. We were particularly interested whether reliable SSVEP responses and oscillatory entrainment can be evoked when (a) the stimulation source is indirectly attended with covert attention and (b) the flickering of the stimulation is below an individually estimated perceptibility threshold. Furthermore, we explored oscillatory entrainment effects of such stimulation protocols in the occipito-frontal(-central) functional cortical network. We found reliable SSVEPs at 10 Hz and the first harmonic response for the perceptible and non-perceptible FM rhythmic stimulation. The SSVEPs could be observed not only when the LED was directly focused but also when participants processed the light source covertly without direct fixation. The absence of an entrainment effect of the B-IPT stimulation in the covert attention condition in participant 14 might be explained by a potentially different behavior compared to the other participants during the condition (also discussed below). When comparing the four stimulation protocols, no meaningful difference in the SNR values, z-scored SSVEP power, and SSVEP amplitudes between the non-perceptible and perceptible rhythmic light stimulation with either overt attention or covert attention could be observed. Previous literature suggests the SSVEP power increases to be a consequence of the entrained neuronal oscillator triggering the resonance peak stimulated at (cf. Dreyer et al., 2017) and not a superimposition of single reactions to each of the repetitive light stimuli (see Capilla et al., 2011, for alternative view in the debate).

The finding of comparable SSVEPs at 10 Hz in all four conditions is contrary to those reported in the literature, especially regarding attention effects on SSVEPs (Walter et al., 2012; Ordikhani-Seyedlar et al., 2014; Dreyer et al., 2017). A major difference to the reported studies was the orientation of the directly and indirectly focused stimuli. While most of the studies positioned the light sources laterally next to each other, we instructed participants to either focus on the LED directly (overt attention condition) or focus on a fixation cross-hair 10 cm below the light source (covert attention condition). A further possible explanation for differences in the attention effects could be the distance between participant and screen as well as fixation point and stimulus. We chose the distances according to those in Dreyer et al. (2017). Walter et al. (2012), however, positioned the participants 10 cm close to the screen and stimulated, therefore, a more eccentric area of the fovea. Thus, comparable SSVEPs for the overt and covert attention condition in our study might be explained by an insufficient distance between the fixation point and stimulation source. Since the stimulation was processed on the edge of the parafoveal vision (5.7°), differences in the oscillatory entrainment between the attention conditions might have been too small to detect via the EEG. Since the participants could freely move their head and eyes, they might have moved their eyes voluntarily or involuntarily toward the flickering light source after the onset and/or during the stimulation in the covert attention condition. Thus, this might have potentially reduced attention effects between the two conditions. We tried to prevent it by instructing the participants in detail, familiarizing them with the experiment in practice runs, and asking them at the end of the experiment if they followed the instruction. None of the participants reported any behavior that deviated from the instructions. By simultaneously using eye-tracking during the experiment, future research could address this limitation.

Interestingly, we observed pronounced power at the harmonic response (20 Hz) in the overt attention condition (Figure 5, left). The strong responses in the harmonic might be explained by non-linear and saturating stimulation effects deforming the EEG signals when fixating directly on the light stimulation. In the covert attention condition, the SSVEP amplitude values were larger for the stimulation below the individual perceptibility threshold than for the stimulation above the threshold. A possible explanation could be that the stimulation in the covert attention condition was directly penetrating the retina on a more eccentric area (around 5.7°), in contrast to the overt attention condition in which the light fell directly on the fovea (0°). Hence, the number of stimulated rods, cones, and the size of the receptive fields of the ganglion cells belonging to these photo-receptors differ between the overt and covert attention condition. Since rods predominate in the peripheral vision, the processing in the peripheral vision is more light sensitive. Although we estimated the IPT separately for the two attention conditions, the rhythmic light stimulation below the individual threshold in the covert attention condition might reveal a stronger modulation due to the higher density of rods and their sensitivity. Subjectively, participants rated the stimulation below the individual threshold in both attention-based conditions as less flickering compared to the stimulation above the individual threshold, but with no difference between the overt and covert attention condition. As fatigue and sleepiness increased over the course of time in the study, the finding of reliable SSVEPs via stimulation protocols aiming at reducing excessive straining of the visual system is promising. In line with Dreyer and Herrmann (2015), the FM stimulation was not rated as clearly flickering even when stimulated above the individual perceptibility threshold with an average response of *M* = 2.61 ± 0.93 in the covert and *M* = 2.52 ± 0.63 in the overt attention condition (on a scale of 1–5).

Regarding our findings of global oscillatory entrainment effects during the four stimulation protocols within the functional connectivity analysis, no significant difference between conditions were observed in the alpha and beta band as well as the alpha and beta peak, respectively. However, Figure 6 indicates a tendency of slightly higher connectivity measures associated with higher coupling between the occipito-frontal(-central) regions for the stimulation below the individual perceptibility threshold. Although non-significant in comparison to the stimulation above the individual perceptibility threshold, the slightly higher coupling between occipital and frontal regions during the B-IPT stimulation might point to simultaneous evaluation processes whether the stimulation is flickering or not (Sauseng et al., 2005; Siegel et al., 2008; Hipp et al., 2011). Results regarding global oscillatory entrainment are especially interesting, since global effects, e.g., in the frontal regions, might be associated with executive functions, inhibitory control, and attention-related processes (Niendam et al., 2012). Future research could investigate further functional networks (e.g., the occipito-parietal network) regarding oscillatory entrainment effects of different stimulation protocols.

### 4.1. Conclusion

To conclude, the finding of robust SSVEPs, even when stimulated below the individual perceptibility threshold and without direct fixation of the stimulation source, reveals the strong potential of rhythmic light stimulation in naturalistic applications like BCIs. A further promising application could be in the context of vehicle interiors (e.g., design of illuminated dashboards) and mobility (e.g., illuminated traffic signs or lights at the car body) to non-intrusively stimulate the brain. A better understanding of effects of the rhythmic light stimulation and influencing factors certainly is a precondition for its integration in out-of-the-lab applications. To ensure a sufficiently long stimulation, we chose a rather long stimulation interval of 8 s. However, future research could investigate the minimum required length of stimulation to evoke reliable SSVEPs and, thus, further reduce eye strain. In addition, inter-individual differences (e.g., in age) on entrainment effects as well as characteristics of individuals who are not sensitive or suitable for rhythmic light stimulation (e.g., the latter is already known in the case of a diagnosed epilepsy, Vialatte et al., 2010) should be explored. Finally, further stimulation frequencies and their entrainment effects in the respective frequency bands (e.g., beta or gamma bands) could be investigated, since this could also prevent interference effects when unintentionally stimulating at the individual alpha frequency. In a next step, we aim at exploring the potential of FM rhythmic light stimulation during simple cognitive and behavioral tasks (e.g., working memory or attention tasks). Such insights regarding effects of the stimulation on performance and attention are important to determine whether rhythmic light stimulation is beneficial to (1) prevent drowsiness during monotonous driving tasks (Hagenmeyer et al., 2007; Ibarra-Orozco et al., 2008), or (2) increase attention before likely transitions (e.g., switching from autonomous to manual driving) in order to improve take-over time, quality and, thus, safety (Melcher et al., 2015; Hirsch et al., 2020; Wörle et al., 2020). We aim at identifying such beneficial effects on attention and performance. Thereby, we will explore stimulation protocols characterized by increased user experience due to decreased eye fatigue and a larger fixation area around the light source as in the case of the stimulation below the individual perceptibility threshold in the covert attention condition.

## Data Availability Statement

The datasets presented in this study can be found in online repositories. The names of the repository/repositories and accession number(s) can be found at: https://osf.io/auvmc/?view_only=5d1711db21d74344b5f1a0dff330b0f5.

## Ethics Statement

The studies involving human participants were reviewed and approved by the Ethics Committee at the Medical Faculty of the Eberhard Karls University and at the University Hospital Tübingen. The patients/participants provided their written informed consent to participate in this study.

## Author Contributions

KL, IS, MW, FD, IP-S, and MV planned the research. Data collection was done by KL, MB, and IS. Data analysis and interpretation was carried out by KL, AD, JR, and MV. KL, AD, IS, MB, MW, FD, JR, and MV prepared the manuscript. All authors contributed to the article and approved the submitted version.

## Conflict of Interest

MW and IP-S were employed by the company Volkswagen AG. The remaining authors declare that the research was conducted in the absence of any commercial or financial relationships that could be construed as a potential conflict of interest.
